# Complaints and the Saturday Fund

**Published:** 1913-02-08

**Authors:** G. A. Lewcock

**Affiliations:** Member of Surgical Appliance Committee, Hospital Saturday Fund. 54 Solon Road, Brixton


### The Editor's Letter-Box.

### Complaints and the Saturday Fund.

*To the Editor of* The Hospital.

Sir,—I note in your issue for February 1 a paragraph referring to the Hospital Saturday Fund, which I welcome most cordially, as it will bring to the notice of the various hospital committees the present dissatisfaction of the Hospital Saturday Fund subscribers respecting the treatment meted out to them on presenting letters from this Fund. This dissatisfaction is not a new thing, but has been gradually getting worse since the matter was brought before the Board of Delegates at the quarterly meeting on July 4, 1908. With all due respect to your informant, his statement hardly represents the case. The main cause of our dissatisfaction is the way in which our subscribers are questioned and insulted by the so-called lady almoner at certain hospitals; and if this continues much longer, the hospitals will have to thank themselves for the loss of the working-men's subscriptions; and it will be striking a great blow at the existence of the Hospital Saturday Fund also. It is quite true that our subscriptions dropped £2,000 last year, and it is hardly to be wondered at, as there has been a number of cases of dissatisfaction during the same period to my own knowledge.

The main question is : Are our subscribers entitled to treatment in respect of the amount subscribed by them to the hospitals? Our articles of association state distinctly that the Fund is empowered :

"4. To give relief, either gratuitously or not, to patients residing in Middlesex, Essex, Kent, Surrey, and Hereford."

And on page 19, Hospital Saturday Fund Report, 1911 : "The Distribution Committee . . . shall also have power to *purchase* from time to time such letters of recommendation as may be required."

The main question here is : Why *purchase*, if there is no consideration for the purchase-money? I willingly allow that the Insurance Act now provides insured persons with medical treatment by the panel doctors, but what about the wives and children? How are they to be treated? I could give you some figures with regard to the number of hospital letters issued by the Hospital Saturday Fund and the amount paid by that Fund for these letters, which would not be pleasant reading for the hospitals, but space forbids. Asking you to give publicity to this communication.—I am, Sir, yours very truly,

G. A. Lewcock,
Member of Surgical Appliance Committee,
Hospital Saturday Fund.

54 Solon Road, Brixton, February 4.

[A reference to this letter will be found in our Notes this week.—Ed. The Hospital.]

